# Untargeted NMR Study of Metabolic Changes in Processing Tomato Treated with *Trichoderma atroviride* Under Open-Field Conditions and Exposed to Heatwave Temperatures

**DOI:** 10.3390/molecules30010097

**Published:** 2024-12-29

**Authors:** Lorenzo Pin, Anatoly Petrovich Sobolev, Giulio Testone, Giuseppe Scioli, Flavia Pinzari, Francesco Magnanimi, Giuseppe Colla, Mariateresa Cardarelli, Donato Giannino

**Affiliations:** 1 Institute for Biological Systems, Italian National Research Council, Monterotondo, 00015 Rome, Italy; lorenzo.pin@isb.cnr.it (L.P.); giulio.testone@cnr.it (G.T.); giuseppe.scioli@isb.cnr.it (G.S.); flavia.pinzari@cnr.it (F.P.); francesco.magnanimi@uniroma1.it (F.M.); 2 Department of Biology and Biotechnology, Sapienza University of Rome, 00185 Rome, Italy; 3 Department of Agriculture and Forestry Science, University of Tuscia, 01100 Viterbo, Italy; giucolla@unitus.it (G.C.); tcardare@unitus.it (M.C.)

**Keywords:** untargeted NMR, metabolic profiles, processing tomato, quality, heatwave, cultivation site, microorganism

## Abstract

Rising temperatures due to climate change may affect the quality of open-field cultivated processing tomatoes by altering the nutrient content. Bioinoculants are growing in popularity as a nature-based strategy to mitigate these environmental stresses. Untargeted quantitative NMR spectroscopy was leveraged to characterize the metabolome of tomato fruits exposed to abiotic stress during the year 2022, which was marked by unexpected high temperatures and low rainfall compared to the year 2021 with average conditions. This study was conducted at growing sites in Tarquinia and Viterbo, comparing untreated plants to ones treated with a *Trichoderma*-based bioinoculant. The hotter year affected the water-soluble fraction (28 compounds), causing an increase in amino acids, citrate, and formate contents while decreasing carbohydrates together with a significant drop in β-sitosterol + campesterol in the organic fraction (11 compounds). The site mainly affected the linolenic acid levels, which were more abundant in Tarquinia than Viterbo in the hotter year, whereas ascorbate and myo-inositol were higher in Tarquinia in both years. The year × site interaction significantly affected the content of several amino acids, glucose, sucrose, and trigonelline. The bioinoculant effect was significant only for sucrose, while its interactions with the other factors showed little to no significance across all the measured metabolites.

## 1. Introduction

A global warming threat to agricultural productivity stands in the rise of average temperatures along with the increasing frequency and intensity of heatwaves [1]. The latter are commonly defined as periods characterized by unusually high and persistent temperatures compared to the seasonal average in a geographical region. Being sessile, plants are vulnerable to temperatures exceeding their tolerance thresholds, leading to a reduction in photosynthesis and disrupted water homeostasis, increasing transpiration and dehydration. High temperatures can also cause protein denaturation and oxidative stress, which trigger signaling pathways and defense mechanisms such as reactive oxygen species scavenging, heat shock proteins, and osmolytes to counteract the stress. Relatedly, the exposure of tomato crops to high temperatures affects several morpho-physiological processes of growth and ultimately product yield and quality [2]. Looking at the processing tomato sector, which is economically relevant all over the world and Italy [3], a 10% loss in products delivered to the canning industry was recorded in 2022 vs. 2021 in Italy due to extreme climatic events [4]. Tomato plants can thrive between 18 °C and 30 °C, and the impact of heat stress on production varies (in extent and type of damage) with the timing of occurrence with respect to growth stages [5]. Briefly, temperatures exceeding optimal ranges (18–25 °C) during outdoor post-transplant can dramatically affect fruit set due to sequential impairments of photosynthesis, aerial organ development, and flower fertility, while excessive heat near or at harvest can alter fruit metabolism and the quality standards (firmness, shelf-life, color) of processing tomatoes [6]. Given the wide use of tomato fruits in diets, numerous studies have been published on the contents of fruit metabolites, including processing cultivars [7], as influenced by agronomic techniques and environmental factors. Moreover, the contribution of bio-active compounds of benefit to human health has been extensively reviewed [8]. As for heat stress’s impact on fruits of sensitive varieties, soluble sugars have been reported to undergo drastic fluctuations, while increased levels of osmolytes, such as proline and putrescine, and the loss of ascorbic acid and lycopene are recorded together with lipid profile re-modeling [9,10]. The accumulation of some amino acids occurs in response to mild heat stress, functioning as a tolerance enhancer. To date, studies on wide pools of amino acids influenced by natural heat stress events are scarce in processing cultivars. Contextually, amino acids’ importance for human health was recently highlighted, and new dietary guidelines enriched in bioactive amino acids were proposed [11].

Protecting open-field production from heatwaves relies on several strategies and technologies, including optimal water management, innovative mulching to minimize soil leakage, and, if possible, adjusting the planting time to avoid peak heat periods [12,13]. The use of improved hybrids [14] relies on genes controlling general stress tolerance or specifically gas exchange performance via stomata conductance/transpiration, via introgression by marker-assisted selection from selected genotypes or wild species [15], or via direct genomic modifications [16]. The use of biostimulants (BSs) and other beneficial microorganisms (BMs), such as *Trichoderma* spp., has gained popularity as a sustainable strategy to mitigate crop stress. BSs comprise diverse substances and/or microorganisms, and their efficacy varies with formulation and environmental conditions, requiring applications tailored to production systems [17]. To date, much research is needed to unravel the mechanisms subtending the interactions between BMs, plant physiology, and the environment to optimize their use for different crops and stress [18]. Common effects/mechanisms induced by BMs include enhanced root development, reduced transpiration (both often regulated by key hormones), and the accumulation of antioxidants and heat shock proteins that protect cell functions from oxidative damage. Moreover, BSs based on extracts or by-products are often degraded by the plant into bioactive signals capable of initiating beneficial processes against stress, so if BS treatments are not synchronous/proximal with the stress, the plant response may turn weak or ineffective [19]. In tomato crops, BSs consisting of arbuscular mycorrhizae showed increased resilience to excessive salinity [20] and induced higher tolerance in heat-stressed tomato plants or mitigated negative effects combined with drought [21]. Non-arbuscular fungi improved tolerance to imposed thermal and water deficit stress, acting on antioxidant metabolism [22,23].

Fungi of the genus *Trichoderma* are among the most widely used biostimulants and biocontrol agents in agriculture [24]. These opportunistic soil fungi can kill other fungi and penetrate plant roots. They are widely employed as biofungicides and inducers of plant defenses against pathogens and abiotic stresses. Their beneficial effects include growth promotion and enhanced stress tolerance, primarily due to intimate root interactions facilitated by secreted proteins that enable root contact, attachment, penetration, and the activation of plant systemic defense [25]. *Trichoderma* species display significant inter- and intraspecific differences in functional traits and ecological roles across soil and other substrates, such as wood or fungi of different taxa. Mycoparasitism is a key trait of these fungi [24]. Among them, *Trichoderma atroviride* is a well-studied biological control agent and a model for research on the regulation of physiological activities, such as asexual sporulation, in response to environmental stimuli like light, mechanical damage, or biotic and abiotic stresses [26]. Focusing on the role of *Trichoderma* spp. in alleviating high-temperature (HT) stress, several key properties have been identified, including proteomic adaptations, where thermotolerant isolates facilitate proteomic changes that enable plants to withstand HT stress [27], biopriming effects, where the *Trichoderma*-induced mitigation of thermal stress in tomato involves the reprogramming of oxidative stress markers and the enhancement of defense networks [28]; antagonistic properties, where some *Trichoderma* isolates inhibit phytopathogens and help maintain plant health under HT stress [29]; and growth promotion, where heat-tolerant *T. harzianum* strains enhance crop growth parameters, such as improving okra resilience to HT [30]. These findings underscore the potential of *Trichoderma* to mitigate HT stress, although challenges remain, including achieving consistent field performance under variable environmental conditions and understanding the long-term impact of these biocontrol agents in diverse agricultural systems.

Multi-omics studies have notably expanded and generated massive data that have been used to understand plant physiological and molecular mechanisms under stress and to develop stress mitigation strategies [31]. Contextually, untargeted NMR analyses of metabolic changes allow the simultaneous investigation of the physiological response of plant organs to stress and product quality (e.g., healthy or unhealthy compounds). Over a two-year study examining the effects of *Trichoderma atroviride* stimulation on processing tomato crops, an unexpected temperature spike during the flowering and fruit-setting stages enabled a detailed evaluation of how site conditions, temperature, and *Trichoderma* treatment interacted to influence fruit metabolic composition. We also investigated whether and to what extent the levels of key nutrients were affected, focusing on stress-responsive markers in the amino acid pool and whether *Trichoderma* treatments compensated for the environmental effects.

## 2. Results

### 2.1. Analyses of Climate Conditions and Main Production Parameters

**Figure 1:**
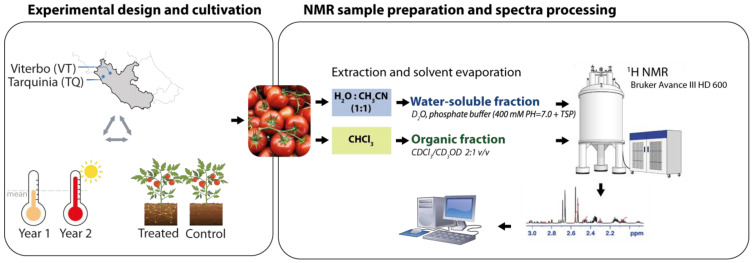
Experimental flow chart. The figure summarizes the variability factors (**left panel**) and the technologies used to produce metabolic profiles (**right panel**).

**Figure 2:**
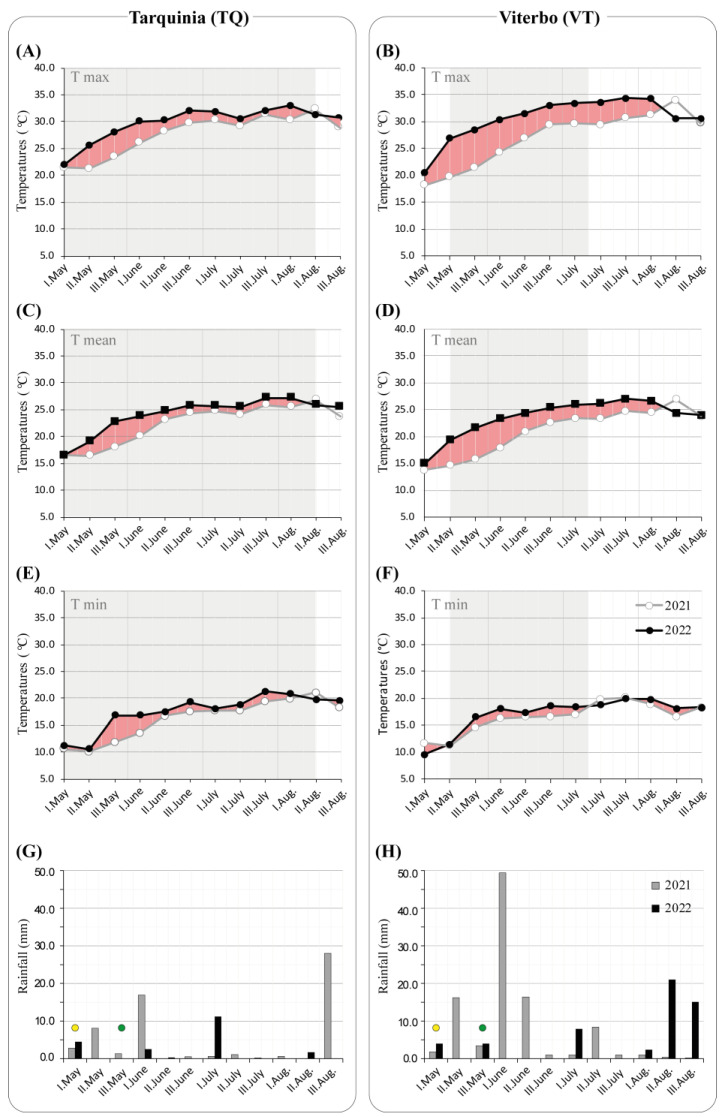
Climate conditions during cultivation in 2021 and 2022. (**A**–**F**) Plots of the maximum, mean, and minimum temperatures (T max, T mean, and T min, respectively) for each decade (I, II, III) during the cultivation months from May to August in 2021 and 2022 for the Viterbo ((**A**,**C**,**E**) panels) and Tarquinia ((**B**,**D**,**F**) panels) sites. Red areas mark positive temperature differences between 2022 and 2021. Grey shading marks periods where T max, T mean, and T min were all higher in 2022 as compared to 2021. (**G**,**H**) Rainfall during the growing season in Viterbo (**G**) and Tarquinia (**H**). *Trichoderma* treatment was done to soil/root system before the transplant (yellow circle) and 15 days after transplant by drip irrigation (green circle).

In this study, the influence of three factors on the content of fruit metabolites in a processing tomato was investigated: the year (factor Y), with 2021 (Y1) and 2022 (Y2) as the levels; the cultivation site (S), including the areas of Viterbo (VT) and Tarquinia (TQ); and the treatment (T) with and without bioinoculant (BI and CN, respectively). The experimental flow has been outlined in Figure 1. In both sites, the cultivation in Y2 was exposed to higher mean temperatures than in Y1, particularly in the May–June period (Figure 2A–F), when flowering and fruit set occur, which are both crucial for production. Moreover, VT experienced 3–5 day-long (short-term) temperatures with values 5 °C higher in Y2 than in Y1. Secondly, both sites (Figure 2G,H) received less cumulative rainfall at the end of the production cycles in Y2 compared to Y1 (TQ, 20.3 vs. 60.1 mm; VT, 54.4 vs. 100.2 mm), especially in the May–June period (TQ, 7.4 vs. 29.6 mm; VT, 8.0 vs. 88.2 mm).

Productivity differed by growing site, with a higher marketable yield in VT (2.72 ± 0.14 kg/plant) than in TQ (1.89 + 0.10 kg/plant), and by growing year, with an average value of 2.89 ± 0.11 kg/plant in Y1 compared with 1.81 ± 0.08 kg/plant in Y2. Moreover, plant mortality associated with the presence of the *Pythium* spp. pathogen was found in 2021, with a lower incidence for *Trichoderma* treatment (about 4% and 2% in TQ and VT, respectively) compared to the controls (about 14% and 8% in TQ and VT, respectively). As for the fruit dry weight (DW) of the samples, the Y factor had the highest significant effects, independently of the treatment and site, and the average in 2022 (4.29 ± 0.50%) was lower than in 2021 (5.47 ± 0.18%) with an esteemed loss of ca. 28%.

### 2.2. Content Variation of the Water-Soluble Fraction

**Figure 3:**
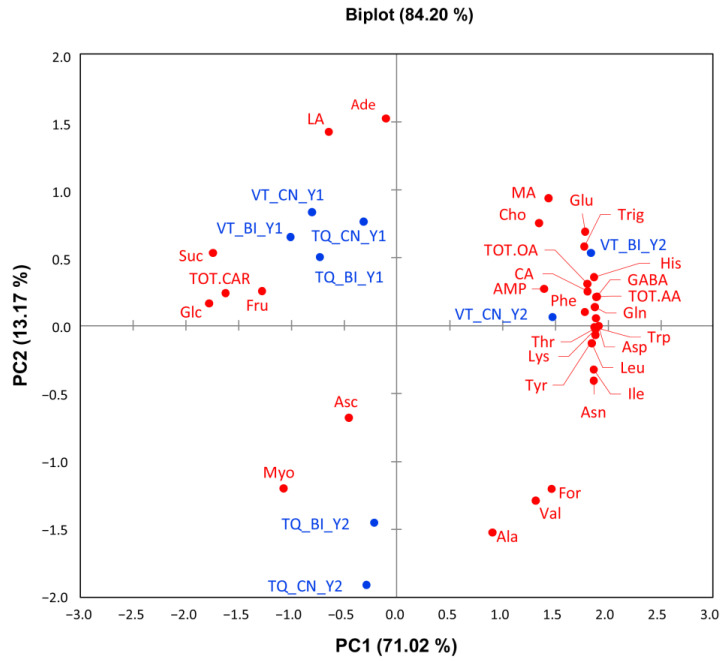
The PCA biplot shows the distribution of 28 hydrosoluble compounds in the tomato fruits from plants bioinoculated (BI) or not (CN) with *Trichoderma* in productive cycles carried out in Viterbo (VT) and Tarquinia (TQ) in the years 2021 (Y1) and 2022 (Y2). The compounds are typed in red, and their abbreviations are in the footnote of Table 1.

An untargeted NMR approach was carried out to assess content variations in the water-soluble (WS) and liposoluble (LS) metabolites of tomato fruits (Figure 1, right panel). Twenty-eight WS compounds were assigned (Table S1 and Figure S1), quantified (Table 1), and grouped into amino acids (*n* = 15), carbohydrates (3), organic acids (5), and others (5). Dataset exploration by PCA (Figure 3) resulted in PC1 and PC2 explaining 71.02% and 13.17% of the total variance. The variables of Y1 fell in the upper left quadrant and were neatly separated from those of Y2, which were in the upper right (VT site) and lower left quadrants (TQ site). The variables with the highest correlation (R^2^ > |0.7|) to PC1 were leucine and isoleucine (positive correlation) and glucose and sucrose (negative), while valine, alanine, and myoinositol were negatively correlated with PC2. PERMANOVA confirmed the strong incidence of Y (R^2^ = 0.29, F = 21.9, *p* = 0.001), as also visualized by PCA, and the Y × S interaction (R^2^ = 0.20, F = 14.8, *p* = 0.001). From the ANOVA results (Table 1), the year and site emerged to affect the compound content variation at high significant levels (*p* < 0.01 and *p* < 0.001, acronym for both HSL), respectively, in 22 and 13 out of 28 metabolites (78.5 and 46.4%), while the bioinoculant treatment did not exert any significant effect except for Suc. Y × S and Y × T also showed highly significant effects on 67.86% (19/28) and 3.57% (1/28) of the compounds, confirming Y as a major source of variation consistently with PCA clustering. S × T and Y × S × T did not show any incidence.

Hereafter, we will report on some major results on metabolite content variation focusing on environmental effects in the hotter year. The recurring thread in the following results is the variation in average contents for metabolite classes, examples of content variation by comparing hotter Y2 vs. Y1 (climate effect), and examples of content changes due to Y × S effects using the relative change content in percentages.

#### 2.2.1. Carbohydrates

The average total carbohydrate range was 255.76–454.58 mg/g DW (Table 1); Y, S, and Y × T showed effects at different significance levels. In detail, Y had a strong, significant effect on all sugars; S had a strong, significant effect on glucose (Gluc) and fructose (Fruc) but not sucrose (Suc); T had a modest effect only on Suc; and Y × S was significant just for Gluc and Suc. Reporting an example taken from the TQ site, the Fruc mean content was higher in fruits from both treated and control plants in Y1 (212.44 ± 27.47 and 259.26 ± 23.91 mg/g DW) than in the hotter Y2 (195.28 ± 15.31 and 189.59 ± 11.87 mg/g DW), and Gluc had a similar pattern that was higher in Y1 (148.95 ± 20.63 and 187.12 ± 16.70 mg/g DW) than in Y2 (147.52 ± 17.23 and 144.03 ± 8.89 mg/g DW). Comparing Y2 vs. Y1, the relative changes in total carbohydrates (Table 2) showed that fruits from control plants from VT and TQ underwent losses of −31.5 and −25.4%, respectively, while those of bio-inoculated plants were −28.15% and −5.45%. The total carbohydrates drop was the result of decreases in Fruc, Gluc, and Suc, with Suc being a majorly affected compound in VT, respectively, with −56.19% and −69.33% in fruits from controls and treated plants, as well as in TQ, with −35.6% and −23.4%.

#### 2.2.2. Amino Acids

The total amino acid mean range (Table 1) was 43.92 ± 11.06–122.14 ± 20.25 mg/g DW, the influence of Y was at HSL on all amino acids, while that of S was limited to 9 out of 15 (Glu, GABA, Gln, Asp, Asn, Lys, Trp, His, and Thr). The Y × S interaction showed HSL on 86.7% (13/15) of amino acids, confirming the greater influence of Y over the other factors on amino acid content changes. Looking at mean values, the most abundant was Gluc (range: 14.68 ± 3.23–47.08 ± 12.84 mg/g DW), which was strongly affected by Y, S, and the Y × S interaction. Comparing fruits from 2022 to 2021 for control and bioinoculated plants in Viterbo (Table 2) the total amino acid gain was, respectively, +145.47% and +178.11%, while in Tarquinia, changes above 13% were not observed (control = −12.79%, treated = +8.35%). In VT, the amino acids showing the highest increase were Lys (+228.01%) and Asp (226.71%) for the control and Tyr (+323.80%) and Asn (+290.08%) for the bio-inoculated samples. In TQ, Val and Ala were the amino acids with the highest variation for both control (+41.41%, +78.25%) and bio-inoculated (+62.41%, +114.80%) samples when comparing 2022 to 2021.

#### 2.2.3. Organic Acids

The organic acid mean total amount varied from 53.65 ± 12.57 to 87.38 ± 17.53 mg/g DW, with citric acid (CA) being the most abundant (44.83 ± 10.88–74.87 ± 16.19) followed by malic acid (5.07 ± 2.75–9.52 ± 2.16) and ascorbic acid (2.47 ± 0.37–3.82 ± 0.71). Lactic acid (LA) and formic acid (FOR) had much lower values (ranging above 0.01 to 0.14 mg/g DW). Y affected CA and FOR contents at high significant levels, and S strongly influenced citric acid and ascorbic acid, while Y × S exerted an HSL effect only on CA. Comparing 2021 vs. 2022, FOR showed the highest relative increase in both VT (+383.65% and +909.62%, in control and bio-inoculated plants, respectively) and TQ (+476.33% and +625.65%) sites. In VT, CA levels increased in fruits from both control and treated plants (+27.19 and 67.01%), while in TQ, they decreased (−9.6%) in the control tomatoes and increased (+16.34%) in treated ones, bringing up the Y × S effect.

#### 2.2.4. Miscellaneous: Trigonelline, Adenosine and Adenosine Monophosphate, Myoinositol, Choline

The average amounts of trigonelline were from 0.09 ± 0.02 to 0.17 ± 0.04 mg/g DW and it was affected at an HSL by Y, S, and Y × S. From 2021 to 2022, remarkable increases of +218.42% and +232.12% were registered in Viterbo for both control and bio-inoculated samples, respectively. In Tarquinia, the decrease was consistent in tomatoes from control plants (−48.47%) and negligible in fruits from treated ones (−3.62%). Adenosine (Ade) ranged from 0.22 ± 0.090 to 0.38 ± 0.02 mg/g DW, while adenosine monophosphate ranged from 1.50 ± 0.21 to 2.49 ± 0.93 mg/g DW, and only Ade was affected by Y and Y × S at high, significant levels. In the 2022 vs. 2021 comparison, the adenosine level decreased in samples from both control and bio-inoculated plants from TQ, though with different intensities (−49.72% and −29.78%, respectively). In fruits from VT, an opposite trend consisted of an increase in tomato samples from controls (+8.70%) and a drop in bio-inoculated plants (−5.50%). The myoinositol levels (1.73 ± 0.53 to 3.19 ± 0.76 mg/g DW) did not undergo any effect by the variability sources, while the choline content (0.54 ± 0.01 to 0.93 ± 0.05 mg/g DW) was affected at an HSL by the Y × T and Y × S interactions and not by the single factors.

### 2.3. Content Variation of the Liposoluble Fraction

**Figure 4:**
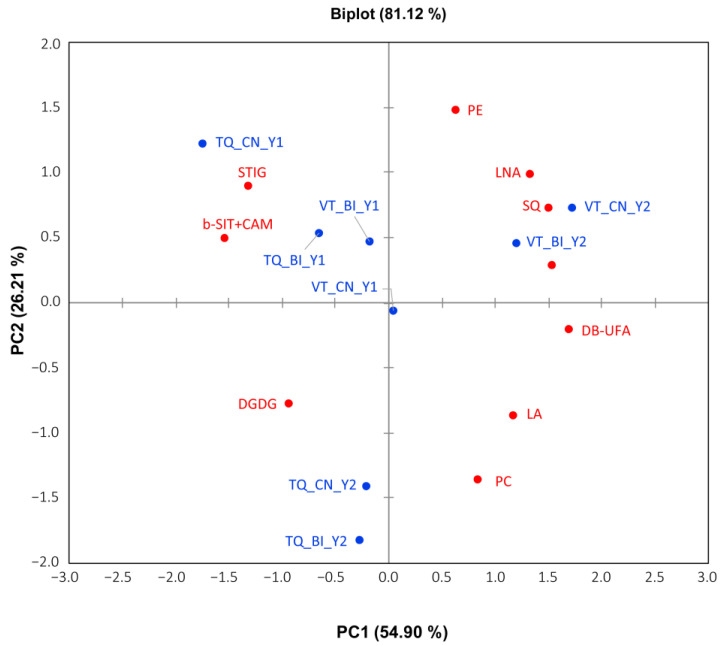
The PCA biplot shows the distribution of 11 liposoluble compounds in tomato fruits from plants bioinoculated (BI) or not (CN) with the *Trichoderma*, in productive cycles carried out in Viterbo (VT) and Tarquinia (TQ) in years 2021 and 2022 (Y1 and Y2). Abbreviations: b-SIT + CAM, β-sitosterol + campesterol; STIG, stigmasterol; SQ squalene; FA, fatty acids; LA, linoleic acid; LNA, linolenic acids; PE phosphatidylethanolamine; PC, phosphatidylcholine, DGDG, digalactosyldiacylglycerol; DB-UFA, all double bonds in unsaturated fatty acids, CH2-allylic UFA, CH2-unsaturated fatty acids.

Liposoluble compounds were assigned by NMR spectra (Table S2 and Figure S2) and quantified as molar percentages (Table 3). The exploratory PCA (Figure 4) consisted of PC1 and PC2, which explained 54.90% and 26.21% of the total variance, respectively, and the distribution of the factors was quite similar to that of the hydrosoluble fraction in Figure 3. Looking at the year of production, all the samples of year 1 (Y1) from the VT and TQ areas fell in the PC1 negative values (except one, VT_CN_Y1, close to zero), together with those of the hotter year (Y2) from TQ, while those of VT of Y2 belonged to the PC1 positive values. In addition, the samples from TQ produced in Y1 and Y2 were distributed on PC2 positive and negative values, respectively. The site effect was visible, as the samples from VT and TQ in Y1 clustered in the upper left quadrant and diverged to the upper right and lower left quadrants for VT and TQ, respectively, in the hotter year Y2. The PERMANOVA results indicated that Y influenced the liposoluble fraction (R^2^ = 0.13, F = 5.64, *p* = 0.001) at a slightly higher significance than S (R^2^ = 0.12, F = 5.31, *p* = 0.001). This datum was confirmed by ANOVA results (Table 3); indeed, Y and S were the only factors affecting content variations at high significant levels (5 out of 11 metabolites for both Y and S, though on different fatty acid types). Relevantly, the *Trichoderma* treatment and all the interactions between factors did not exert effects at HSL (*p* < 0.05 are encountered for some compounds in Y × S, T × S, and Y × T × S combinations). In the table below, we report the results based on the same criteria described for the hydrosoluble fraction.

#### 2.3.1. Sterols and Squalene

The total amounts of β-sitosterol and campesterol (b-SIT + CAM) ranged from 2.23 ± 0.55 to 4.19 ± 0.43 mol%, and only Y affected them at HSL. Referring to the 2022 vs. 2021 comparison (Table 4), a general trend of diminishment (below −22%) characterized both treated and control plants in both cultivation sites, with higher drops in VT (−40.34% and −32.18%). The stigmasterol (STIG) levels ranged from 2.33 ± 0.43 to 3.54 ± 0.49 mol%, (Table 3). None of the factors affected the contents, though the values of relative changes based on means pointed at decremental trends by comparing the two years. Squalene (SQ), which is a terpene and a precursor of sterols, was observed to vary between 3.82 ± 0.67 and 7.92 ± 1.31 mol%, and only S affected its content at HSL. As for the relative content variation in 2022 vs. 2021 (Table 4), remarkable increases in squalene levels were recorded in tomatoes from Viterbo (over 62%), while a modest decrease occurred in Tarquinia (up to −7.14%).

#### 2.3.2. Fatty Acids

The molar percentages of linoleic acid (LA) ranged from 44.14 ± 3.13 to 37.00 ± 1.65 mol%, and the single factors showed no effect, except for a modest influence in the triple interaction (Table 3). The linolenic acid (LNA) contents ranged from 5.01 ± 0.84 to 9.81 ± 2.12 mol%, and the variation was only influenced by S at HSL, with trends of increases and decreases in VT and TQ. LA and LNA belong to di- and tri-unsaturated fatty acid groups (DUFA and TUFA); the number of double bonds in unsaturated fatty acids (DB-UFA) levels varied between 90.20 ± 6.75 and 107.13 ± 1.75 mol%; Y and S affected their contents with HSL. The range of the CH2-allylic UFA group (154.05 ± 8.10 and 184.51 ± 21.75 mol%) was affected just by the year at HSL. Comparing 2022 vs. 2021, their decrease occurred in both control (16.17%) and treated (9.88%) samples from Viterbo, while a different trend was recorded in those from the respective control (2.04%) and treated (4.13%) ones from Tarquinia.

#### 2.3.3. Phospho- and Galacto-Lipids

Phosphatidylethanolamine (PE) levels were between 3.15 ± 1.79 and 5.72 ± 1.20 mol%; those of phosphatidylcholine (PC) were 9.32 ± 0.92 and 13.18 ± 2.83 mol%. The relative amounts of digalactosyldiacylglycerol (DGDG) varied from 2.92 ± 0.66 and 3.72 ± 0.74 mol%. No statistical significance was observed for these compounds. Regarding PC, by comparing samples of 2021 vs. 2022, a trend of increased contents was recorded in both sites.

### 2.4. Correlation Analysis

**Figure 5:**
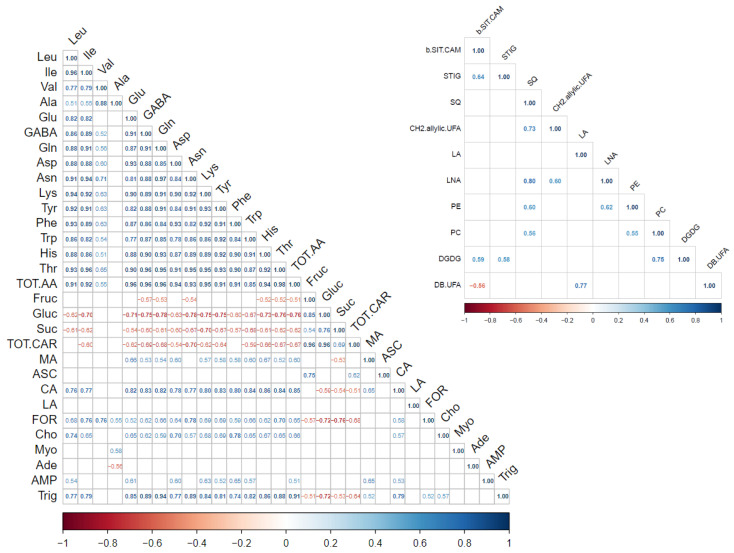
Corrplots of hydrosoluble (**left**) and liposoluble (**right**) metabolite contents in tomato fruits. Correlation classes for *p* ≤ 0.01: strong, r ≥ |0.70|; mild, |0.50| ≤ r ≤ |0.69|; low, r ≤ |0.49|.

A correlation analysis was first developed using all metabolites to bring up relationships; however, those between hydrosoluble and liposoluble fractions were of modest significance. Below, we propose two correlograms, each per distinct group (Figure 5), and we chose to report the results by selecting events of strong correlation defined by r ≥ |0.70| and *p* ≤ 0.01.

## 3. Discussion

### 3.1. Overview

The analysis of climate data confirms that 2022 was warmer than 2021 by +0.82 °C for Italy [32], and it was the hottest year (+1.15 °C) compared to the 1991–2020 period (www.isac.cnr.it/climstor/DPC/climate_news.html, accessed on 27 December 2024). A decade-based analysis of May and June temperatures at both cultivation sites (Figure 2) shows prolonged periods of extreme minimum and maximum temperatures in 2022, which were 3–5 °C higher than those in 2021. This trend was also confirmed for the broader VT area [33]. In June 2022, the extreme temperature values were 5.49 °C and 3.02 °C above the 2004–2021 mean, indicating heatwaves as defined by the European State of the Climate 2023 [34] (https://climate.copernicus.eu/sites/default/files/custom-uploads/ESOTC%202023/Summary_ESOTC2023.pdf, accessed on 27 December 2024). Regarding the metabolic changes in tomatoes, as influenced by climate, soil, and *Trichoderma* treatments, untargeted NMR analysis showed that the climate and cultivation site were the primary sources of variation. Although few studies have specifically investigated the effects of high temperatures on tomato fruit in open-field conditions [35], our results offer new insights. The discussion below will focus on the variations in compound content (primarily determined by NMR), emphasizing temperature and site-specific effects, as well as the influence of *Trichoderma atroviride* application.

### 3.2. The Hydro Soluble Fraction

#### 3.2.1. Carbohydrates

The glucose and fructose contents quantified by NMR in tomato fruit vary with the genotype and ripening stage. In previously analyzed genotypes, the Gluc levels ranged from 700 to 2200, and the Fruc levels ranged from 1200 to 2500 mg/100 g FW [32]. At harvest, Gluc was reported to be between 1000–2200 and Fruc was between 1500–3000 mg/100 g FW [33]. In this study, the ranges (conversion factors are in Table 1 legend) were lower for both carbohydrates (Gluc 460–913 and Fruc 672–1112 mg/100 g FW), while the higher levels of Fruc vs. Gluc were maintained consistently. Fruc was also quantified by NMR in distinct tomato berry tissues [34], and the sum values of meso-endo-mesocarp fractions (230–250 mg/g DW) were consistent with the values from this work. Finally, the Suc content by NMR at 500 and 700 MHz [35] varied from 5–14 and 3–9 µmol/g FW, which was higher than here (0.3–1.3 µmol/g FW). Sugars and amino acids are among the most responsive metabolites (in terms of content variation) to environmental changes and exert impact on tomato organoleptic [36] traits. Moreover, tomato fruit affected by heat stress showed reduced fruit soluble sugar content due to the higher activity of sucrose-metabolizing enzymes [37]. This aligns with the lower carbohydrate levels observed in the hotter 2022 season, likely caused by reduced carbohydrate import due to leaf photosynthesis impairment, as reported for tomatoes under heat stress [38].

#### 3.2.2. Amino Acids

The measured ranges are consistent with the NMR profiles of amino acid contents reported in ripened tomato fruits from various cultivars [32], in which Glu, Gln, and GABA were the most abundant (100–450; 40–110; 20–90 mg/100 g FW; here: 88–257; 55–142; 11–202 mg/100 g FW), followed by Asn, Asp, and Ala (15–25; 15–45; 15–90 mg/100 g FW; here: 13–40; 38–62; 7–13 mg/100 g FW). Increases in these amino acids normally occur with ripening [33,39]. Moreover, significant changes in Glu, Gln, and GABA depended on cultivation sites [40], as observed in this work. Focusing on fruits from Viterbo, the accumulation of total amino acids occurred in fruits from both control and treated plants (+145 and +178%), suggesting a great incidence of stress in the hotter 2022. Relatedly, the Gln, Glu, GABA, and Asn increases (from +139 to +290%) suggested a general response for stress protection and were consistent with the rise in the same amino acids in mature tomatoes of salt-stressed plants [41]. Lys, Tyr, and Thr (affected by Y, S, and Y × S) also showed a gain of over 200% in the hotter year; Lys and Tyr are, respectively, precursors of polyamines and phenylpropanoids and known to participate in stress responses [42,43], while a Thr increase is reported in heat-stressed tomato fruits as being directly involved in the synthesis of heat shock proteins or indirectly supporting the glutathione-related antioxidant defense [9].

The contents of Ile, an essential branched-chain amino acid (BCAA), and Phe were significantly affected by Y and Y × S, with an over 100% increase observed in fruits from VT in 2022. Ile has assessed roles in jasmonic acid signaling, which triggers the heat shock protein (HSP) to refold and stabilize proteins damaged by high temperatures [44], while Phe mitigates oxidative stress in abiotic stress by reducing ROS buildup [45]. In this work, Val and Ala showed a gain trend (from +20 to +137%) independently of site and treatment (ANOVA confirmed the unique influence by the year). Val has known roles in osmoprotection in drought tolerance [46] and shares similar functions to Ile (both BCAA) in HSP synthesis [47]. Ala accumulates in heat stress events [48] and specifically against hypoxia [49], which would not be the case in our investigations into tomato fruits. Val can derive from pyruvate; however, the absent correlation with sugar contents suggests that its accumulation is due to proteolytic events rather than biosynthesis. This is supported by evidence showing that, in drought-stressed Arabidopsis, Val accumulates as a result of protein degradation mediated by ABA [46]. Finally, Pro metabolism in heat-stressed plants is characterized by rapid cycles of accumulation and degradation in fruits [50]. Here, the Pro content could not be detected as being below the NMR threshold detection level, likely due to the fruit stage.

The carbohydrate and amino acid contents in tomato fruits were the most sensitive to environmental changes among various metabolites [40]; here, a significant negative relationship between the lowering of glucose and the rising of several amino acids was observed in fruits. The reduction in fruit sugars may result from altered transport from stressed leaves, where impaired photosynthesis and increased respiration cause a decrease in carbon fixation and starch accumulation. This is supported by omics studies in tomato leaves exposed to heat and salt stress [51]. However, studies indicate that fruits specifically respond to heat stress through intense sugar catabolism, which promotes amino acid synthesis [52] and through protein degradation [53], both of which elevate the amino acid spool to counteract stress. Finally, considering berry quality, nutritional features are expected to vary due to the increase of essential amino acids (Lys, Ile, Leu, Met, Val, Phe, Trp) as well as taste characteristics due to the rise in Glu, which is responsible for “umami”, or of Asn and Trp, which are positively related to sweetness in tomato cultivars [54].

#### 3.2.3. Organic Acids

The contents of citric, malic, and formic acids were measured by NMR [32,33] and shown to vary with tomato cultivars (300–450; 7–25; 0.7–1.1 mg/100 g FW) and fruit stage (200–380; 10–38; 0.05–0.35 mg/100 g FW), and ranges in this work were consistent (241–317, 21–41, 0.1–0.4 mg/100 g FW). Those of lactate were sensibly minor (0.04–0.8 vs. 5.0–25 mg/100 g FW) and poorly, if at all, influenced by all source variation factors; lactate is a fermentation marker (of microbial or fruit origin), and high contents spoil the quality of processing tomatoes [55]. The ascorbic acid content by HPLC in industrial tomatoes ranged from 150 to 280 mg/kg FW among diverse cultivars, with complex patterns during ripening [56]. These values were not dissimilar to those measured here (131–153 mg/kg FW), as influenced by the site only. Heat stress affects the metabolism of organic acids in tomatoes, specifically influencing the citric and malic contents through various interactions between environmental factors and fruit physiology [37]. It is often reported that citric acid amounts increase while the malic acid level fluctuates, which is not unexpected considering their roles in the tricarboxylic acid cycle as branchpoint metabolites directing various pathways. The formic acid contents of tomato fruits varied from 0.05 to 1.1 mg/100 g FW based on the ripening stage and diverse cultivars [32,33] and were consistent with ranges measured in this study (0.1–0.4 mg/100 g FW), which were also strongly and significantly influenced by the year. For instance, a gain of mean contents of 300 up to 900% was shared by all fruits in the hotter 2022, independently of site and treatment. Formic acid can derive from the methanol-formaldehyde route, the serin-glycine pathway, or tryptophan (Trp) degradation via the kynurenine pathway (poorly characterized in plants), and the strong correlation between Trp and formic acid suggests that the latter pathway might contribute to defense in heat stress.

#### 3.2.4. Miscellaneous

The discussion on adenosine (Ade), adenosine monophosphate, myoinositol, and choline, with the latter three being minimally—if at all—influenced by the variation sources, is restricted to content ranges, which were generally consistent with the literature on tomato. Briefly, Ade and adenosine diphosphate (ADP) contents were previously reported in the range of 1.4–1.7 and 4.0–9.0 mg/100 g FW, respectively [33], while in this study, their levels were found to be 0.8–2.1 mg/100 g FW for Ade and 8.2–10.7 mg/100 g FW for AMP. Myo levels, determined by GC/MS, were previously measured at 90–300 mg/g FW [57], aligning with our findings of 73–133 mg/g FW. Finally, Cho was reported at 7.2–9.4 mg/100 g FW [33], which are slightly higher values than those measured here (2.3–4.0 mg/100 g FW). Focussing on trigonelline, our findings show values strongly influenced by the year and site, ranging between 2.3 and 4.0 mg/100 g FW and consistent with previously reported NMR-based ranges of 1.1–3.3 mg/100 g FW [32,33]. The high content gain (over 200%) recorded in TQ during the heatwave year and the strong correlation with tryptophan (r = 0.82, *p* ≤ 0.01)—which is a trigonellin precursor in the kynurenine pathway—support that Trig accumulation may be a response to stress events. This is in addition to its established role as an osmoprotectant, as it shifts from 0.8 to 1.7 mg/g DW (measured by NMR) when the water potential decreases in drought-affected tomato leaves [58].

### 3.3. The Liposoluble Fraction

Overall, year and site only had a highly significant influence on sterols (b-SIT + CAM), squalene (SQ), and unsaturated fatty acid groups (Table 3), while the *Trichoderma* treatment did not have any. We will mainly discuss content ranges and their variation in relation to the year effect in cultivation affected by heatwaves. Moreover, we will give some speculation on positive correlations between SQ vs. linolenic acid (LNA) and allylic unsaturated fatty acids (UFA) and neglect some others, in which a component represents a fraction of the total (linoleic acid/UFA) or not affected by the factors (DGDG/PC).

#### 3.3.1. Sterols and Squalene

Sito- and stigma-sterol contents quantified by NMR and expressed in molar percentages [32,33] were reported to vary with cultivars and ripening stage (respectively, 3.1–5.9 and 1.8–3.5 mol%), showing consistency with the ranges of b-SIT + CAM measured here (3.3–6.3 mol%), while those of STIG were higher (3.6–5.3 mol%). Squalene is a triterpene and a key precursor of sterols. In mature tomato fruits, the mean ratios between SQ and sterol contents (as quantified by GC-MS/HPLC) ranged from 1 to 4 [59]. In this work, however, the higher ranges of SQ (11.4–23.7 mol%) than sterols may derive from differences in analytical methodology or from Y and S effects. However, the increase in SQ content (plus 62–86%) in the hotter year in Viterbo was concomitant with a decrease in sterols (minus 32–40%), suggesting that stress conditions may trigger SQ accumulation to support membrane stability or oxidative metabolism. As for SQ/LNA relationships, it is reported that during tomato ripening (chloroplast-to-chromoplast transition), SQ levels tend to rise, while linolenic contents decrease [60]. Here, we can speculate that the positive correlation between squalene and linolenic is indirect; specifically, SQ may be produced as a compensatory response to the oxidation of LNA under heat stress. The strong positive correlation of SQ with the highly reactive allylic unsaturated fatty acids further supports this hypothesis.

#### 3.3.2. Fatty Acids

In the literature, the levels of DUFA (24.7–30.7 mol%) and TUFA (8.9–15.9 mol%) varied with tomato genotypes [32] and were in the ranges of linoleic and linolenic acid contents of this work. The year and site affected UFA and allylic UFA, with particular significance for the hotter year, during which relevant content gains occurred regardless of site and treatment. Consistently, in the literature, heat-induced molecular signatures from tomato fruit evidenced changes in the degree of membrane lipid unsaturation [9].

#### 3.3.3. Phospho- and Galacto-Lipids

The levels of phosphatidylethanolamine (PE) and phosphatidylcholine (PC) are phospholipids and key components of cell membranes and integrity, they vary with ripening and storage conditions in tomato berries [61]. The PE fraction is usually much lower than the PC [62], which was consistent with the data of this work. Galactolipids are predominant in plastid membranes, and their content decreases with green-to-red ripening and storage and is lower than phospholipids [61], as was observed in this work for digalactosyldiacylglycerol.

### 3.4. On the Bioinoculant Effects

Statistical analyses revealed a mild significant effect (*p* ≤ 0.05) of the *Trichoderma* treatment on the sucrose content of tomato fruits as a single ANOVA factor (Table 1). A slightly lower sucrose content was observed in berries from treated plants compared to the control group. Limited statistical significance was also noted for fructose, choline, and citric acid concentrations when considering the interaction between year and treatment. Specifically, the fructose, choline, and citric acid concentrations were higher in fruits from control plants compared to treated plants in the first year, whereas the opposite trend was observed in the second year of experimentation. Fungi of *Trichoderma* spp. have been reported to enhance tolerance to heat and water deficit stress by triggering anti-oxidant defense [22,23]. While these effects were not observed at a highly significant level in this study, the observed metabolic variation trends suggest potential activation of antioxidant and osmolyte pathways (e.g., choline production) in response to elevated temperatures in 2022 [63]. Expanding the focus of analysis to include a broader metabolite spectrum or targeting different developmental stages prior to harvest could help better delineate the effects of *Trichoderma* treatments. Previous studies have shown that *T. atroviride* enhances the availability of nitrogen and phosphorus in the soil [64], promoting nutrient uptake and increasing root and shoot biomass [65]. This greater availability of nutrients, coupled with the fungus’s biocontrol properties, likely contributed to the observed differences between treatments, particularly under the extreme conditions of 2022. For example, under higher temperatures, *Trichoderma* may have mitigated stress-related metabolic changes by improving nutrient assimilation and offering protection from biotic stressors. Investigating macro- (N, P, K, Ca, Mg, S) and microelements (Fe, Mn, Zn, Cu, Cl, etc.) uptake as well as pathogen suppression effects could further clarify the role of *T. atroviride*. While this study did not directly address soil-microbiota interactions or rhizosphere dynamics, future research should prioritize these aspects to understand the broader implications of *Trichoderma* inoculation. Additionally, the effects of the bioinoculant might be more pronounced under less extreme or more stable environmental conditions, such as those observed in the first experimental year when temperature and humidity values were within historical averages. This is supported by the improved post-transplant survival rates recorded under these conditions, emphasizing the potential for *Trichoderma* to enhance plant resilience in standard cultivation scenarios. Finally, while the observed effects of *Trichoderma* treatment on metabolic changes were limited in significance, the trends noted in this study highlight the potential of this bioinoculant to influence stress-related pathways and nutrient assimilation under challenging environmental conditions. Further targeted analyses and experimental designs will be necessary to fully elucidate its mechanisms and optimize its use in agricultural systems.

## 4. Materials and Methods

### 4.1. Plant Growth, Conditions and Yield

Tomato seedlings (*Solanum lycopersicum* L.) of hybrid “Perfectpeel” F1 (Bayer-Seminis Vegetables Italia, Milan, https://www.vegetables.bayer.com/it, accessed on 27 December 2024) at the three true leaves stage were transplanted in paired rows, with a 0.38 m spacing along the rows and 0.40 m among the rows. The distance on the row was 0.36 m and between bines of 1.70 m with a planting density of 3.2 plants/m^2^. The treatment with *Trichoderma atroviride* (P. Karst., Bidr. Känn., Synonyms: *Hypocrea atroviridis* Dodd, Lieckf. and Samuels, *Hypocreaceae, Hypocreales, Hypocreomycetidae, Sordariomycetes, Ascomycota*) was carried out 48 h before transplant by dipping the seedlings up to the collar in an aqueous solution of the fungus powder formulation (1.6 × 10^7^ CFU/seedling) for 20 min [66]. Plantlets underwent a second treatment (3.1 × 10^7^ CFU/plant) by drip irrigation 15 days after field transplant. Two production cycles were carried out in 2021 and 2022 (ca. 110-day cycle, transplant to harvest, May to August) and in parallel at two experimental farms, “ARSIAL” (www.arsial.it/dove-siamo/, accessed on 27 December 2024) in Tarquinia (42°13′ N, 11°43′ E; 22 m a.s.l.) and “Nello Lupori” (University of Tuscia, www.unitus.it/ateneo/strutture-e-servizi/cia/azienda-agraria-didattico-sperimentale-nello-lupori/, accessed on 27 December 2024) in Viterbo (42°25′ N; 12°08′ E; 310 m a.s.l.). The soil of ARSIAL farm is classified [67] as loamy-sandy-clay (48% sand, 18% silt, and 34% clay) with low organic matter content (1.17%); pH is 6.8, cation exchange capacity (CEC) is 19.9 meq/100 g, and salinity is 0.151 dS/m. Moreover, total nitrogen is 0.077%, and exchangeable cations (mg/kg) are as follows: K (617), P (57), Ca (2820), Mg (299), Na (83). The soil of “Nello Lupori” farm is sandy loam (67% sand, 18% silt, and 15% clay) with moderate organic matter content (1.80%); pH is 7.1, CEC is 27.7 meq/100 g, and total nitrogen is 0.087%; exchangeable cations (mg/kg) are as follows: K (1470), P (21), Ca (3100), Mg (250), Na (104). In both trials, irrigation was carried out with a drip irrigation system with in-line emitters located 0.30 m lines apart and an emitter flow rate of 3.4 L h^–1^. Drip lines were located along each row. Total irrigation volume was 3.200 and 2.750 m^3^/ha for ARSIAL and “Nello Lupori”, respectively. Plant pathogens and pests were controlled in both trials with four foliar treatments of copper-based fungicide (Cupravit Bio Advanced; Bayer Crop Science, Milano, Italy) at a rate of 1.5 kg/ha each and two foliar treatments of deltamethrin-based insecticide (Decis Evo; Bayer Crop Science, Milano, Italy) at a rate of 0.5 kg/ha each.

### 4.2. Experimental Design and Sampling

A total of 8 randomized plots (4 for treatment and 4 for control) were employed and set with 3.6 plants/m2 density (plot size: 18 m × 12 m; 3 rows/plot; 20 plants/row; row spacing: 1.7 m × 0.36 m). For each plot, berries (average weight 65 ± 3 g) of the third branch of randomly selected plants (n = 5) were collected and bulked in sterile plastic bags, stored in darkened polystyrene boxes, and shredded in labs on the same day. Specifically, the fruits’ surfaces were cleaned with paper towels wetted with sterile water and gently dried, then chopped and immediately frozen in liquid nitrogen, ground to powder by pestle and mortar, and stored at −80 °C. The samples (8 g) were weighed without thawing, lyophilized at −50 °C for 72 h (FreeZone^®^ 4.5 Liter Freeze Dry Systems Labconco, Kansas City, MO, USA, 7740030 shelved model), and stored at −20 °C. A bulk of 10 tomatoes (replicate bulks, RB) represented an experimental plot; hence, the analyses were carried out using quadruple RBs.

### 4.3. Extraction of Hydro- and Liposoluble Metabolites, Assignment and Quantification

As for the hydrosoluble (synonym water-soluble, WS) fraction, lyophilized and cold (−20 °C) material (50 mg) was added to 1.7 mL of acetonitrile/water (1:1 *v*/*v*) into 2.2 mL tubes, the mixture was vortexed for 30 s, centrifuged for 5 min (5000× *g*), and the supernatant (1.5 mL) was recovered and stored on ice; a total of 1.5 mL of acetonitrile/water (1:1 *v*/*v*) was added to the pellet for a second round of extraction as described above. The supernatants were pooled, and a final vol of 3 mL was evaporated by nitrogen flux at room temperature (RT), and the residue was dissolved in 0.75 mL of 400 mM phosphate buffer (pH = 7) in deuterated water plus the internal standard 3-(trimethylsilyl)-propionic-2,2,3,3-d4 acid sodium salt (TSP) at a concentration of 1 mM. The NMR spectra of the WS compounds were recorded on a Bruker AVANCE III HD 600 NMR spectrometer (Bruker, Billerica, MA, USA, 27 °C, proton frequency 600.13 MHz); the internal standard for the ^1^H spectrum was given by the signal of the TSP methyl group (δ = 0.00 ppm), each spectrum was acquired by co-addition of 256 transients with a recycle delay of 7 s, residual HDO signal was suppressed by pre-saturation, and the whole procedure was performed using a 45° pulse of 7.0–5.5 µs, 32 K data points. The spectra were further processed using Bruker TOPSPIN software (version 3.6), and after Fourier transformation, manual and baseline corrections allowed the selection of resonances in the ^1^H NMR spectra (Table S1, Figure S1), which were integrated to calculate the metabolite concentrations, using the integral value of the TSP methyl groups (9H) as a reference for quantification. Integral ranges for each signal are reported in Table S1. Metabolite content was expressed as mg g^−1^ dry weight. Overall, 28 hydrosoluble metabolites were assigned and quantified by NMR using the methodology reported previously [32].

As for the liposoluble (LS) fraction, 50 mg of lyophilized material was added to 0.6 mL of methanol/chloroform (2:1 *v*/*v*) and vortex-shaken for 30 s; the mixture was consequentially added with chloroform (0.2 mL) + shaking (30 s) and distilled water + shaking (0.24 mL + 30 s), stored at 4 °C for 40 min, and then centrifuged for 15 min (4200× *g*) at 4 °C. The upper hydro-alcoholic phase was discarded, and the lower organic phase was accurately recovered (0.50 mL) and stored in a glass vial. The above-described procedure was repeated using the same pellet and halved volumes of solvents to finally recover 0.25 mL of extract, which was pooled with the first one (final vol 0.75 mL) and fully evaporated by nitrogen flux at RT, and the dry residue was dissolved in 0.75 mL of deuterated chloroform/methanol mixture (2:1 *v*/*v*). The NMR spectra of the LS compounds were recorded on the same apparatus as above (27 °C, proton frequency 600.13 MHz); the internal standard for the ^1^H spectrum was given by the signal of the tetramethylsilane methyl group (δ = 0.00 ppm). Each spectrum was acquired by co-addition of 256 transients with a recycle delay of 4 s; residual OH signal from methanol was suppressed by pre-saturation, and the whole procedure was performed using a 90° pulse of 8.0 µs, 32 K data points. For spectra of LS fraction (Figure S2), the integrals (I) of 11 selected signals numbered from I_1_ to I_11_ were measured (Table S2) and normalized versus the integral of -CH_2_ groups of all fatty acid chains (I_5_). The contents of fatty acids, sterols, and lipids were expressed as molar percentages (mol%) using the listed equations (1 to 10): b-SIT + CAM = 2·I_1_/3I_5_ (1); STIG = 2·I_2_/3I_5_ (2); SQ = 2I_3_/6I_5_ (3); LA = 100I_6_/I_5_ (4); LNA = 100 I_7_/2I_5_ (5); PE = 100I_8_/I_5_ (6); PC = 2I_9_/9I_5_ (7); DGDG = 200I_10_/I_5_ (8); DB-UFA = 100I_11_/I_5_ (9); CH_2_-allylic UFA = 100I_4_/I_5_ (10).

### 4.4. Statistical Analyses

Raw NMR data of HS and LS compounds were loaded into R (version 4.3.3) and tested for normality by the Shapiro–Wilk test. Data for principal component analysis (PCA) were scaled to mean zero and standard deviation 1. Permutational Analysis of Variance (PERMANOVA) was carried out on the Euclidean distance matrix to further infer results visualized by the PCA by the |adonis2| function of R |vegan| package (v 2.6-8). Finally, |aov()| function was used to perform three-way ANOVA. We accounted for multiple testing by applying the Bonferroni correction to the *p*-values. Fresh and dry (lyophilized) weights of fruits were recorded and tested for differences among Y, T, and S through t-tests and ANOVA after data normalization. The |psych| package (v 2.4.3) was used to inspect potential relationships among hydro- and liposoluble metabolites; briefly, two datasets were merged into one, the |corr.test()| function created the correlation matrix among all the metabolites, the Pearson correlation coefficients and the adjusted *p*-values (Benjamini-Hochberg procedure) were produced, and the results were pictured in a correlation plot by using |corrplot| package (v 0.92).

## 5. Conclusions

In this study, we investigated the complex interactions among climate change, cultivation site conditions, and the application of a *Trichoderma*-based bioinoculant, focusing on their combined effects on the metabolic profile of tomato fruits. High temperatures during the second year of experiments significantly influenced the levels of key metabolites, particularly carbohydrates, amino acids, and organic acids. Among the carbohydrates, fructose and glucose showed marked reductions, consistent with the effects of heat stress on photosynthesis and carbohydrate transport known in the literature. In contrast, amino acids such as GABA, Glu, Gln, Lys, and Phe accumulated, highlighting their role in plant stress response and temperature tolerance. Similarly, organic acids exhibited significant changes influenced by cultivation site and temperature, suggesting their critical role in maintaining physiological and metabolic homeostasis under stress. This study underscores the importance of cultivation practices and soil amendments in shaping the metabolome of tomato fruits, with temperature effects able to outweigh *Trichoderma*-specific impacts. These findings provide insights into the effects of environmental stress and biostimulant applications on tomato biochemical composition, offering valuable perspectives for enhancing crop quality and resilience under future climate scenarios. Further research should explore the genetic, environmental, and agronomic interactions necessary to support sustainable agricultural systems.

## Figures and Tables

**Table 1:** Content variations of hydrosoluble compounds (mg/g DW) in fruits from *Trichoderma*-treated and control plants over two production years and significance from three-way ANOVA test.

Compounds	Y1 (2021)				Y2 (2022)				ANOVA						
	VT		TQ		VT		TQ		Significance						
	BI	CN	BI	CN	BI	CN	BI	CN	Y	T	S	YxT	YxS	TxS	YxSxT
Fruc	184.80 ± 25.65	195.68 ± 27.16	212.44 ± 27.47	259.26 ± 23.91	158.98 ± 15.04	156.85 ± 9.19	195.28 ± 15.31	189.59 ± 11.87	***	n.s.	***	*	n.s.	n.s.	n.s.
Gluc	163.05 ± 21.206	172.00 ± 23.49	148.95 ± 20.63	187.12 ± 16.70	94.29 ± 13.35	96.92 ± 12.54	147.52 ± 17.23	144.03 ± 8.89	***	n.s.	***	n.s.	***	n.s.	n.s.
Suc	8.12 ± 2.06	8.45 ± 0.85	6.20 ± 0.85	8.21 ± 1.81	2.49 ± 0.77	3.71 ± 0.68	4.75 ± 1.87	5.28 ± 0.98	***	*	n.s.	n.s.	**	n.s.	n.s.
TOT.CAR	355.97 ± 45.98	376.14 ± 51.36	367.59 ± 48.2	454.58 ± 38.92	255.76 ± 27.89	257.47 ± 21.05	347.55 ± 29.19	338.9 ± 21.29	***	n.s.	***	*	n.s.	n.s.	n.s.
Leu	0.22 ± 0.04	0.23 ± 0.07	0.27 ± 0.04	0.36 ± 0.05	0.54 ± 0.08	0.46 ± 0.10	0.33 ± 0.08	0.33 ± 0.03	***	n.s.	n.s.	n.s.	***	n.s.	n.s.
Ile	0.15 ± 0.04	0.15 ± 0.06	0.19 ± 0.04	0.25 ± 0.02	0.43 ± 0.07	0.41 ± 0.10	0.25 ± 0.08	0.27 ± 0.05	***	n.s.	n.s.	n.s.	***	n.s.	n.s.
Val	0.15 ± 0.05	0.19 ± 0.06	0.22 ± 0.03	0.26 ± 0.03	0.36 ± 0.06	0.34 ± 0.07	0.35 ± 0.13	0.37 ± 0.09	***	n.s.	n.s.	n.s.	n.s.	n.s.	n.s.
Ala	1.29 ± 0.61	1.87 ± 1.16	1.35 ± 0.22	1.70 ± 0.23	2.47 ± 0.65	2.25 ± 0.36	2.90 ± 1.55	3.03 ± 1.21	***	n.s.	n.s.	n.s.	n.s.	n.s.	n.s.
Glu	16.65 ± 4.21	17.56 ± 7.49	17.72 ± 6.31	23.22 ± 4.17	47.08 ± 12.84	41.04 ± 10.24	16.33 ± 4.69	14.68 ± 3.23	***	n.s.	***	n.s.	***	n.s.	n.s.
GABA	2.26 ± 1.09	2.39 ± 0.62	2.18 ± 0.98	2.76 ± 0.41	7.59 ± 2.03	7.02 ± 2.10	2.96 ± 1.26	2.41 ± 0.68	***	n.s.	***	n.s.	***	n.s.	n.s.
Gln	11.01 ± 2.91	10.93 ± 5.15	12.19 ± 2.32	15.12 ± 0.93	32.24 ± 5.95	33.44 ± 8.62	12.13 ± 2.04	14.76 ± 3.67	***	n.s.	**	n.s.	***	n.s.	n.s.
Asp	7.00 ± 2.16	7.85 ± 2.75	8.04 ± 1.71	9.79 ± 1.49	14.42 ± 2.56	13.8 ± 1.65	8.90 ± 1.55	8.90 ± 0.93	***	n.s.	***	n.s.	***	n.s.	n.s.
Asn	2.36 ± 0.40	2.89 ± 1.38	3.00 ± 0.47	3.49 ± 0.24	9.22 ± 1.81	9.42 ± 2.70	4.49 ± 1.17	4.87 ± 1.25	***	n.s.	***	n.s.	***	n.s.	n.s.
Lys	0.30 ± 0.03	0.27 ± 0.07	0.41 ± 0.07	0.54 ± 0.05	1.06 ± 0.22	0.90 ± 0.19	0.48 ± 0.05	0.49 ± 0.03	***	n.s.	***	n.s.	***	n.s.	n.s.
Tyr	0.18 ± 0.04	0.22 ± 0.14	0.33 ± 0.05	0.37 ± 0.02	0.74 ± 0.23	0.68 ± 0.25	0.34 ± 0.08	0.36 ± 0.05	***	n.s.	*	n.s.	***	n.s.	n.s.
Phe	0.86 ± 0.20	0.89 ± 0.42	1.12 ± 0.19	1.51 ± 0.11	2.03 ± 0.43	1.78 ± 0.27	1.19 ± 0.27	1.16 ± 0.14	***	n.s.	n.s.	n.s.	***	n.s.	n.s.
Trp	0.33 ± 0.08	0.33 ± 0.09	0.30 ± 0.04	0.39 ± 0.03	0.70 ± 0.21	0.63 ± 0.17	0.46 ± 0.12	0.36 ± 0.06	***	n.s.	**	n.s.	**	n.s.	n.s.
His	0.61 ± 0.18	0.57 ± 0.28	0.56 ± 0.08	0.68 ± 0.04	1.21 ± 0.26	1.09 ± 0.22	0.62 ± 0.08	0.60 ± 0.03	***	n.s.	**	n.s.	***	n.s.	n.s.
Thr	0.56 ± 0.16	0.62 ± 0.23	0.66 ± 0.15	0.88 ± 0.08	2.06 ± 0.51	1.95 ± 0.45	0.86 ± 0.31	0.88 ± 0.17	***	n.s.	***	n.s.	***	n.s.	n.s.
TOT.AA	43.92 ± 11.06	46.93 ± 17.76	48.51 ± 11.65	61.29 ± 6.62	122.14 ± 20.25	115.2 ± 26.99	52.56 ± 10.08	53.45 ± 4.25	***	n.s.	***	n.s.	***	n.s.	n.s.
CIT	44.83 ± 10.88	53.72 ± 3.84	44.98 ± 3.65	55.43 ± 1.65	74.87 ± 16.19	68.32 ± 10.58	52.33 ± 6.28	50.11 ± 2.69	***	n.s.	**	*	**	n.s.	n.s.
MAL	6.26 ± 2.06	7.15 ± 2.06	6.22 ± 0.77	6.58 ± 0.98	9.52 ± 2.16	7.88 ± 0.43	5.07 ± 2.75	6.26 ± 0.82	n.s.	n.s.	*	n.s.	*	n.s.	n.s.
ASC	2.47 ± 0.37	2.78 ± 0.61	3.35 ± 0.68	3.82 ± 0.71	2.81 ± 0.35	2.65 ± 0.13	3.37 ± 0.67	3.41 ± 0.48	n.s.	n.s.	***	n.s.	n.s.	n.s.	n.s.
FOR	0.01 ± 0.00	0.02 ± 0.03	0.01 ± 0.00	0.02 ± 0.01	0.09 ± 0.01	0.10 ± 0.02	0.08 ± 0.01	0.09 ± 0.01	***	n.s.	n.s.	n.s.	n.s.	n.s.	n.s.
LAC	0.07 ± 0.05	0.14 ± 0.10	0.07 ± 0.03	0.08 ± 0.01	0.08 ± 0.12	0.01 ± 0.03	0.02 ± 0.02	0.02 ± 0.01	*	n.s.	n.s.	n.s.	n.s.	n.s.	n.s.
TOT.ORG	53.65 ± 12.57	63.79 ± 5.64	54.63 ± 3.6	65.92 ± 2.81	87.38 ± 17.53	78.96 ± 10.77	60.88 ± 8.21	59.88 ± 1.72	**	n.s.	**	*	**	n.s.	n.s.
Cho	0.55 ± 0.08	0.65 ± 0.16	0.75 ± 0.16	0.85 ± 0.06	0.93 ± 0.05	0.81 ± 0.09	0.72 ± 0.11	0.54 ± 0.01	n.s.	n.s.	n.s.	**	***	n.s.	n.s.
Myo	2.31 ± 0.52	2.03 ± 0.77	2.33 ± 0.42	2.85 ± 0.50	1.76 ± 0.53	1.90 ± 0.41	2.71 ± 1.68	3.19 ± 0.76	n.s.	n.s.	*	n.s.	n.s.	n.s.	n.s.
Ade	0.28 ± 0.08	0.27 ± 0.08	0.31 ± 0.07	0.38 ± 0.02	0.27 ± 0.02	0.29 ± 0.03	0.22 ± 0.09	0.19 ± 0.04	**	n.s.	n.s.	n.s.	**	n.s.	n.s.
AMP	1.56 ± 0.29	1.50 ± 0.21	1.71 ± 0.23	2.16 ± 0.37	2.49 ± 0.93	1.85 ± 0.17	1.86 ± 0.42	1.65 ± 0.56	n.s.	n.s.	n.s.	n.s.	*	n.s.	n.s.
Trig	0.14 ± 0.03	0.16 ± 0.03	0.13 ± 0.04	0.17 ± 0.04	0.46 ± 0.12	0.50 ± 0.17	0.12 ± 0.02	0.09 ± 0.02	***	n.s.	***	n.s.	***	n.s.	n.s.

VT and TQ, Viterbo and Tarquinia sites; BI, plants bio-inoculated with *Trichoderma*; CN, untreated plants. n.s. non-significant; *, **, *** = significant at *p* ≤ 0.05, 0.01 and 0.001, respectively. Mean dry weights in Y1 and Y2 were 5.47 and 4.29%, respectively; the conversion factors in mg/g FW to use for dividing the tabulated values are 18.28 and 23.31. Carbohydrates: Fruc, fructose; Gluc, glucose; Suc, sucrose; TOT.CAR, total carbohydrates. Amino acids (AA): Leu, leucine; Ile, isoleucine; Val, valine; Thr, threonine; Tyr, tyrosine; Trp, tryptophane; Ala, alanine; Gln, glutamine; Glu, glutamic acid; Asp, aspartic acid; Asn, asparagine; Phe, phenylalanine; His, histidine; GABA, gamma-aminobutyric acid; Lys, lysine; TOT.AA, total amino acids. Organic acids (OA): CA, citric acid; MA, malic acid; ASC, ascorbic acid; FOR, formic acid; LA, lactic acid. TOT.OA, total organic acids. Various: Cho, choline; Myo, myoinositol; Ade, adenosine; AMP, adenosine monophosphate; Trig, trigonelline.

**Table 2:** Relative content variation of hydrosoluble compound in tomato fruits from treated and control plants.

Compounds	Control (Y2 vs. Y1)			Treated (Y2 vs. Y1)		
	VT	TQ	t	VT	TQ	t
Fruc	−19.85%	−26.87%	↓↓	−13.97%	−8.08%	↓↓
Gluc	−43.65%	−23.03%	↓↓	−42.17%	−0.96%	↓↓
Suc	−56.19%	−35.64%	↓↓	−69.33%	−23.44%	↓↓
TOT.CAR	−31.55%	−25.45%	↓↓	−28.15%	−5.45%	↓↓
Leu	103.23%	−9.31%	↑↓	140.85%	24.59%	↑↑
Ile	176.94%	11.97%	↑↑	200.91%	29.54%	↑↑
Val	78.93%	41.41%	↑↑	137.96%	62.41%	↑↑
Ala	20.45%	78.25%	↑↑	91.66%	114.80%	↑↑
Glu	133.74%	−36.76%	↑↓	182.80%	−7.83%	↑↓
GABA	193.90%	−12.68%	↑↓	235.82%	35.74%	↑↑
Gln	206.08%	−2.41%	↑↓	192.77%	−0.50%	↑↓
Asp	75.74%	−9.07%	↑↓	106.11%	10.65%	↑↑
Asn	226.71%	39.71%	↑↑	290.08%	49.84%	↑↑
Lys	228.01%	−10.26%	↑↓	248.76%	16.79%	↑↑
Tyr	213.22%	−0.09%	↑↓	323.80%	4.64%	↑↑
Phe	101.18%	−23.04%	↑↓	134.93%	6.54%	↑↑
Trp	92.10%	−9.37%	↑↓	112.62%	51.44%	↑↑
His	89.06%	−11.60%	↑↓	100.25%	9.33%	↑↑
Thr	216.43%	−0.31%	↑↓	269.00%	30.49%	↑↑
TOT.AA	145.47%	−12.79%	↑↓	178.11%	8.35%	↑↑
CA	27.19%	−9.60%	↑↓	67.01%	16.34%	↑↑
MA	10.17%	−4.90%	↑↓	52.06%	−18.43%	↑↓
ASC	−4.54%	−10.82%	↓↓	13.73%	0.78%	↑↑
FOR	383.65%	476.33%	↑↑	909.62%	625.65%	↑↑
LA	−91.17%	−73.63%	↓↓	12.01%	−67.56%	↑↓
TOT.OA	23.77%	−9.16%	↑↓	62.88%	11.44%	↑↑
Cho	26.13%	−36.42%	↑↓	68.61%	−4.18%	↑↓
Myo	−6.70%	12.04%	↑↓	−23.86%	16.46%	↓↑
Ade	8.70%	−49.72%	↑↓	−5.50%	−29.78%	↓↓
AMP	23.06%	−23.83%	↑↓	59.32%	9.15%	↑↑
Trig	218.42%	−48.47%	↑↓	232.12%	−3.62%	↑↓

The relative content variation (calculated as the difference in mean values normalized against the year with average temperatures) was expressed as incremental (+) or decremental (−) percentage. VT and TQ, Viterbo and Tarquinia sites. t, trend; upward and downward arrows indicate, respectively, content gain and loss referring to VT and TQ columns in fruits from treated or control plants. Green shade highlights when the same gain or loss patterns occur in fruits from both control and treated plants, independently of the site.

**Table 3:** Content variations of liposoluble compounds (mol%) in fruits from *Trichoderma*-treated and control plants from two production years, and significance from three-way ANOVA test.

Compounds	Y1 (2021)				Y2 (2022)				ANOVA						
	VT		TQ		VT		TQ		Significance						
	BI	CN	BI	CN	BI	CN	BI	CN	Y	T	S	YxT	YxS	TxS	YxTxS
b-SIT + CAM	3.29 ± 0.78	3.75 ± 0.88	3.76 ± 1.05	4.18 ± 0.43	2.23 ± 0.55	2.24 ± 0.46	2.92 ± 0.64	2.80 ± 0.31	***	n.s.	*	n.s.	n.s.	n.s.	n.s.
STIG	2.66 ± 0.88	2.53 ± 0.61	3.25 ± 1.49	3.54 ± 0.5	2.40 ± 0.50	2.53 ± 0.58	2.78 ± 0.76	2.33 ± 0.43	n.s.	n.s.	n.s.	n.s.	n.s.	n.s.	n.s.
SQ	4.55 ± 1.87	4.24 ± 1.80	4.38 ± 1.86	4.02 ± 0.55	7.42 ± 3.35	7.92 ± 1.31	4.07 ± 1.35	3.82 ± 0.67	*	n.s.	**	n.s.	*	n.s.	n.s.
CH2-allylic UFA	155.35 ± 6.84	158.82 ± 3.62	156.52 ± 5.41	154.05 ± 8.10	170.70 ± 11.61	184.51 ± 21.75	162.99 ± 5.05	157.19 ± 6.10	**	n.s.	*	n.s.	*	n.s.	n.s.
LA	40.58 ± 0.75	44.14 ± 3.13	41.00 ± 1.68	37.00 ± 1.65	42.70 ± 4.46	42.44 ± 2.74	41.55 ± 3.93	43.18 ± 2.06	n.s.	n.s.	n.s.	n.s.	n.s.	n.s.	*
LNA	6.97 ± 1.37	6.38 ± 1.31	6.17 ± 1.39	6.55 ± 1.35	8.52 ± 2.59	9.81 ± 2.12	6.37 ± 2.13	5.01 ± 0.84	n.s.	n.s.	**	n.s.	*	n.s.	n.s.
PE	5.24 ± 1.70	5.32 ± 1.66	5.26 ± 1.68	4.66 ± 1.68	5.05 ± 1.65	5.72 ± 1.20	3.69 ± 1.89	3.15 ± 1.79	n.s.	n.s.	n.s.	n.s.	n.s.	n.s.	n.s.
PC	49.68 ± 14.09	51.07 ± 15.99	49.80 ± 14.56	41.97 ± 4.12	50.36 ± 12.02	54.07 ± 10.71	59.33 ± 12.73	55.23 ± 5.97	n.s.	n.s.	n.s.	n.s.	n.s.	n.s.	n.s.
DGDG	1.48 ± 0.42	1.50 ± 0.44	1.59 ± 0.41	1.78 ± 0.25	1.46 ± 0.33	1.57 ± 0.18	1.86 ± 0.37	1.72 ± 0.31	n.s.	n.s.	n.s.	n.s.	n.s.	n.s.	n.s.
DB-UFA	98.64 ± 3.65	101.12 ± 1.82	97.71 ± 2.61	90.20 ± 6.75	103.66 ± 5.55	107.13 ± 1.75	100.10 ± 6.34	98.61 ± 4.69	**	n.s.	**	n.s.	n.s.	*	n.s.

VT and TQ, Viterbo and Tarquinia sites; BI, plants bio-inoculated with *Trichoderma*; CN, untreated plants. n.s. non-significant; *, **, *** = significant at *p* ≤ 0.05, 0.01 and 0.001, respectively. Abbreviations: b-SIT + CAM, ß-sitosterol + campesterol; STIG, stigmasterol; SQ squalene; CH2-allylic UFA, CH2-unsaturated fatty acids; LA, linoleic acid; LNA, linolenic acids; PE phosphatidylethanolamine; PC, phosphatidylcholine; DGDG, digalactosyldiacylglycerol; DB-UFA, all unsaturated fatty acids.

**Table 4:** Relative content variation of liposoluble compound in tomato fruits from treated and control plants.

Compounds	Control (Y2 vs. Y1)			Treated (Y2 vs. Y1)		
	VT	TQ	t	VT	TQ	t
b-SIT + CAM	−40.27%	−33.01%	↓↓	−32.22%	−22.34%	↓↓
STIG	0.00%	−34.18%	-↓	−9.77%	−14.46%	↓↓
SQ	86.79%	−4.98%	↑↓	63.08%	−7.08%	↑↓
CH2-allylic UFA	16.18%	2.04%	↑↑	9.88%	4.13%	↑↑
LA	−3.85%	16.70%	↓↑	5.22%	1.34%	↑↑
LNA	53.76%	−23.51%	↑↓	22.24%	3.24%	↑↑
PE	7.52%	−32.40%	↑↓	−3.63%	−29.85%	↓↓
PC	5.87%	31.59%	↑↑	1.37%	19.14%	↑↑
DGDG	4.67%	−3.37%	↑↓	−1.35%	16.98%	↓↑
DB-UFA	5.94%	9.32%	↑↑	5.09%	2.45%	↑↑

Metabolite abbreviations are listed in Table 3 footnote. Green shade highlights when the same gain or loss patterns occur in fruits from both control and treated plants, independently of the site.

## Data Availability

The raw data supporting the conclusions of this article will be made available by the authors on request.

## Supplementary Materials

The following supporting information can be downloaded at: https://www.mdpi.com/article/10.3390/molecules30010097/s1. Table S1. Summary of water-soluble metabolites identified in 600 MHz ^1^H NMR spectra in tomato fruit extracts dissolved in D_2_O/phosphate buffer. Integral range for quantitativeanalysis is also reported. Table S2. Summary of metabolites identified in 600 MHz ^1^H NMR spectra in tomato fruit organic extract dissolved in CDCl3/CD3OD (2:1 *v*/*v*). Figure S1. ^1^H NMR spectrum of the water-soluble fraction of tomato sample. Solvent: D2O-phosphate buffer (400 mM, pH = 7). Selected signals: 1, Leu; 2, Ile; 3, Val; 4, Thr; 5, Lactic acid; 6, Ala; 7, Glu; 8, GABA; 9, Gln; 10, Citric acid; 11, Asp; 12, Asn; 13, Lys; 14, Choline; 15, Myoinositol; 16, Fructose; 17, Malic acid; 18, Ascorbic acid; 19, β-Glucose; 20, α-Glucose; 21, Sucrose; 22, Tyr; 23, Phe; 24, Trp; 25, His; 26, Adenosine; 27, Formic acid; 28, AMP; 29, Trigonelline. Figure S2. ^1^H NMR spectrum of the organic fraction of tomato sample. Solvent: CDCl3/CD3OD2:1 *v*/*v*. Selected signals: 1, β-Sitosterol + Campesterol; 2, Stigmasterol; 3, Squalene; 4, CH2 allylic; 5, α-CH2 all fatty acids; 6, Linoleic acid, bis-allylic; 7, Linolenic acid, bis-allylic; 8, PE; 9, PC; 10, Digalactosyldiacylglycerol; 11, Double bonds in unsaturated fatty acids.
