# Supplementary material for: Untargeted NMR Study of Metabolic Changes in Processing Tomato Treated with Trichoderma atroviride Under Open-Field Conditions and Exposed to Heatwave Temperatures
Source: Molecules. 2024 Dec 29;30(1):97. doi: 10.3390/molecules30010097 (PMC11721353; doi:10.3390/molecules30010097)
Supplement: Supplementary file 1 [file molecules-30-00097-s001.zip › molecules-3335762-supplementary.pdf]

Supplementary materials

Untargeted NMR Study of Metabolic Changes in Processing Tomato Treated with *Trichoderma atroviride* Under Open-Field Conditions and Exposed to Heatwave Temperatures

Lorenzo Pin [1,†], Anatoly Petrovich Sobolev [1,*,†] , Giulio Testone [1] , Giuseppe Scioli [1] , Flavia Pinzari [1] , Francesco Magnanimi [1,2] , Giuseppe Colla [3] , Mariateresa Cardarelli [3] and Donato Giannino [1,*]

[1] Institute for Biological Systems, Italian National Research Council, Monterotondo, 00015 Rome, Italy; lorenzo.pin@isb.cnr.it (L.P.); giulio.testone@cnr.it (G.T.); giuseppe.scioli@isb.cnr.it (G.S.); flavia.pinzari@cnr.it (F.P.); francesco.magnanimi@uniroma1.it (F.M.)
[2] Department of Biology and Biotechnology, Sapienza University of Rome, 00185 Rome, Italy [3] Department of Agriculture and Forestry Science, University of Tuscia, 01100 Viterbo, Italy; giucolla@unitus.it (G.C.); tcardare@unitus.it (M.C.)
[*] Correspondence: anatoly.sobolev@cnr.it (A.P.S.); donato.giannino@cnr.it (D.G.)
[†] These authors contributed equally to this work.

Table S1. Summary of water-soluble metabolites identified in 600 MHz ^{1}H NMR spectra in tomato fruit extracts dissolved in D_2O/phosphate buffer. Integral range for quantitative analysis is also reported.

^{1}H, ppm	Compound[a]	Integral range	
		From (ppm)	To (ppm)
0.96	Leucine (Leu)	0.982	0.949
1.02[b]	Isoleucine (Ile)	1.028	1.014
1.05	Valine (Val)	1.058	1.030
1.49	Alanine (Ala)	1.509	1.470
2.06	Glutamic acid (Glu)	2.102	2.024
2.30	Gamma-aminobutyric acid (GABA)	2.310	2.282
2.46	Glutamine (Gln)	2.488	2.431
2.81	Aspartic acid (Asp)	2.845	2.782
2.89	Asparagine (Asn)	2.918	2.856
3.04	Lysine (Lys)	3.049	3.038
6.91	Tyrosine (Tyr)	6.936	6.895
7.43	Phenylalanine (Phe)	7.461	7.413
7.74	Tryptophan (Trp)	7.756	7.723
8.22	Histidine (His)	8.240	8.200
1.34	Threonine (Thr)	1.355	1.320
4.02	Fructose (Fruc)	4.054	3.985
5.25	α-Glucose (Gluc)	5.283	5.217
4.66	β-Glucose (Gluc)	4.695	4.620
5.42	Sucrose (Suc)	5.437	5.401
2.55	Citric acid (CA)	2.586	2.516
4.29	Malic acid (MA)	4.322	4.287
4.53	Ascorbic acid (ASC)	4.539	4.520
8.46	Formic acid (FOR)	8.469	8.460
1.35	Lactic acid (LA)	1.355	1.320
3.21	Choline (Cho)	3.220	3.195
3.31	*Myo*-Inositol (Myo)	3.313	3.302
8.36	Adenosine (Ade)	8.367	8.352
8.58	Adenosine-monophosphate (AMP)	8.579	8.551
8.85	Trigonelline (Trig)	8.867	8.829

Notes: [a] abbreviation is in parenthesis; [b] only half of doublet was integrated.

Table S2 Summary of metabolites identified in 600 MHz ^1H NMR spectra in tomato fruit organic extract dissolved in $CDCl_3/CD_3OD$ (2:1 v/v).

	^1H, ppm	Integral range		Group	Number of equivalent protons	Abbreviation	Compounds
		From (ppm)	To (ppm)				
I_1	0.69	0.707	0.680	CH_3	3	b-SIT+CAM	beta-Sitosterol + Campesterol
I_2	0.72	0.731	0.707	CH_3	3	STIG	Stigmasterol
I_3	1.69	1.704	1.667	CH_3	6	SQ	Squalene
I_4	2.04	2.135	1.950	CH_2	4^a	CH_2-allylic UFA	Allylic CH_2 in all unsaturated fatty acids and squalene
I_5	2.32	2.391	2.245	CH_2	2		alpha-CH_2 all fatty acids
I_6	2.77	2.800	2.745	CH_2	2	LA	Linoleic acid, bis-allylic
I_7	2.82	2.845	2.800	CH_2	4	LNA	Linolenic acid, bis-allylic
I_8	3.12	3.156	3.112	CH_2	2	PE	Phosphatidylethanolamine
I_9	3.23	3.250	3.206	$N(CH_3)_3$	9	PC	Phosphatidylcholine
I_{10}	4.91	4.933	4.884	CH	1	DGDG	Digalactosyldiacylglycerol
I_{11}	5.37	5.429	5.301	CH=CH	2^b	DB-UFA	Double bonds in unsaturated fatty acids

Notes: [a] in fatty acid chains; [b] for each double bond

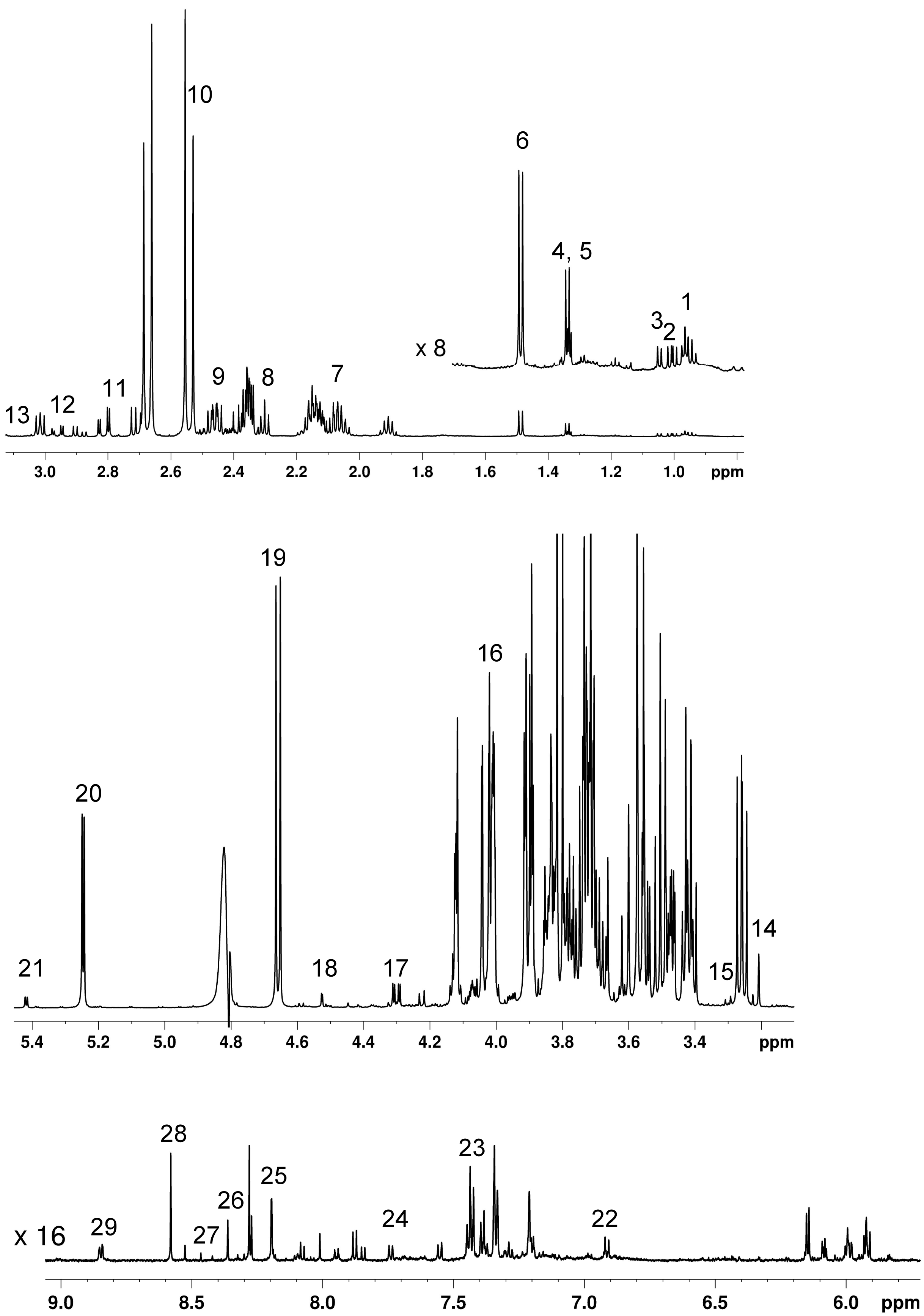

Figure S1. ^{1}H NMR spectrum of the water-soluble fraction of tomato sample. Solvent: D_2O - phosphate buffer (400 mM, pH = 7). Selected signals: 1, Leu; 2, Ile; 3, Val; 4, Thr; 5, Lactic acid; 6, Ala; 7, Glu; 8, GABA; 9, Gln; 10, Citric acid; 11, Asp; 12, Asn; 13, Lys; 14, Choline; 15, *Myo*-Inositol; 16, Fructose; 17, Malic acid; 18, Ascorbic acid; 19, β-Glucose; 20, α-Glucose; 21, Sucrose; 22, Tyr; 23, Phe; 24, Trp; 25, His; 26, Adenosine; 27, Formic acid; 28, AMP; 29, Trigonelline.

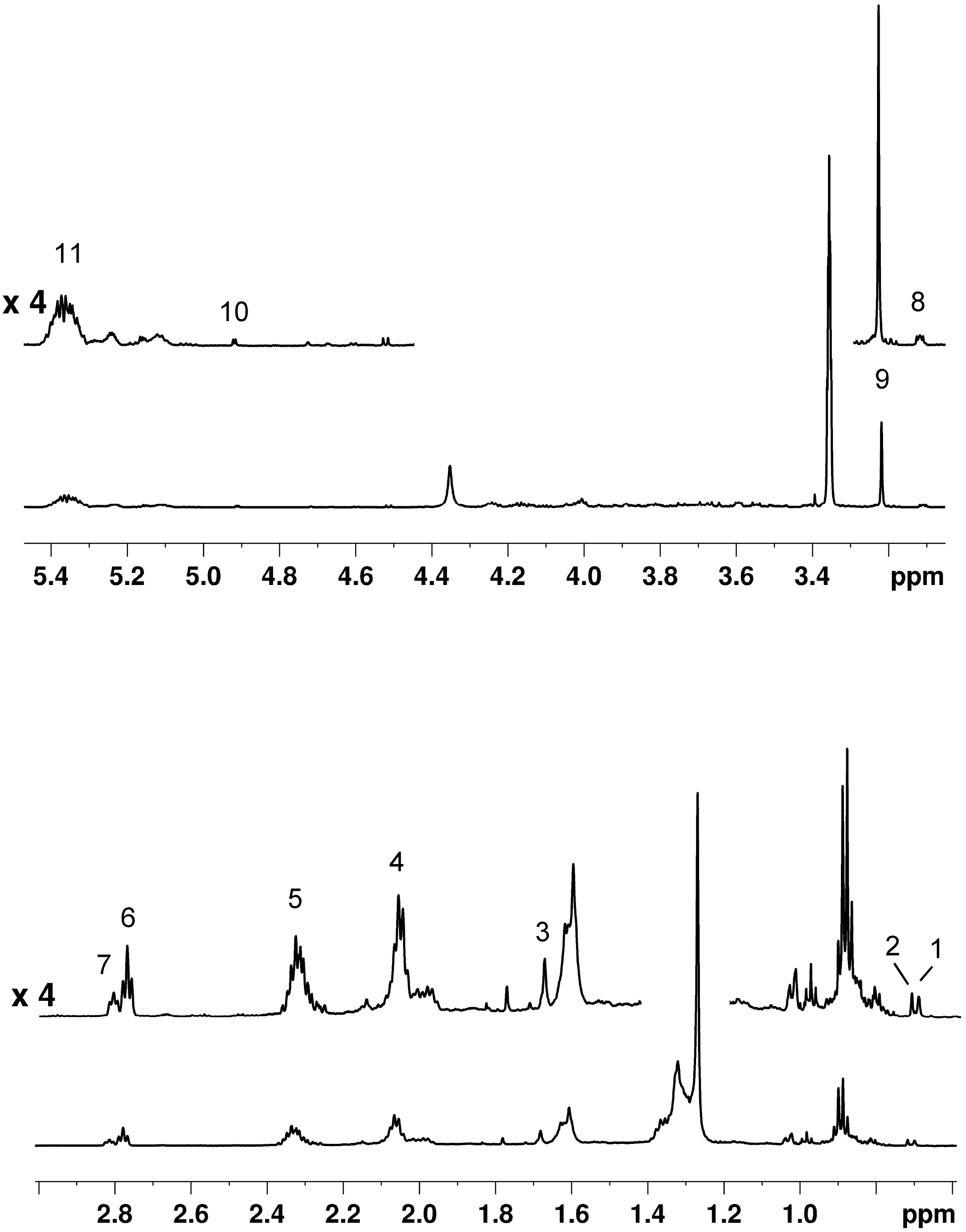

Figure S2. ^1H NMR spectrum of the organic fraction of tomato sample. Solvent: $CDCl_3/CD_3OD$ 2:1 v/v. Selected signals: 1, β-Sitosterol + Campesterol; 2, Stigmasterol; 3, Squalene; 4, CH_2 allylic; 5, α-CH_2 all fatty acids; 6, Linoleic acid, bis-allylic; 7, Linolenic acid, bis-allylic; 8, PE; 9, PC; 10, Digalactosyldiacylglycerol; 11, Double bonds in unsaturated fatty acids.
